# The pituitary gland in SARS-CoV-2 infections, vaccinations, and post-COVID syndrome

**DOI:** 10.1016/j.clinsp.2022.100157

**Published:** 2022-12-22

**Authors:** Josef Finsterer, Fulvio Alexandre Scorza

**Affiliations:** a Neurology & Neurophysiology Center, Vienna, Austria; b Disciplina de Neurociência, Universidade Federal de São Paulo/Escola Paulista de Medicina (UNIFESP/EPM), São Paulo, SP, Brazil

**Keywords:** CNS, Central Nervous System, COVID, coronavirus disease, SARS-CoV-2, severe, acute, respiratory syndrome - coronavirus type-2

## Introduction

There is ample evidence that SARS-CoV-2 not only affects the lungs but all other organs as well, particularly the Central Nervous System (CNS) and the peripheral nervous system (neuro-COVID).^1^ There is also evidence accumulating that any of the SARS-CoV-2 vaccines carries the risk of side effects, including neurological adverse reactions.^2^ A CNS compartment frequently less noticed compared with other CNS structures is the pituitary gland. However, an increasing number of reports demonstrated that the pituitary gland cannot only be involved in SARS-CoV-2 infections but can be also a target of adverse reactions to SARS-CoV-2 vaccinations.^3^ Since the pathophysiology of long-COVID (subacute COVID-19, post-COVID syndrome) remains elusive, it can be speculated that subclinical or mild clinical affection of the pituitary gland is involved in the pathophysiology of post-COVID syndrome. This narrative review aimed at summarising and discussing previous and recent findings regarding the involvement of the pituitary gland in SARS-CoV-2 infections, SARS-CoV-2 vaccinations, and in long-COVID syndrome.

## Methods

A literature search in the databases PubMed and Google Scholar was conducted using the search terms “pituitary gland”, “hypophysitis”, “pituitary apoplexy”, and “neuro-COVID” in combination with “SARS-CoV-2″, “COVID-19″, “coronavirus”, and “long-COVID”. Additionally, reference lists were checked for further articles meeting the search criteria. Included were only original articles detailing individual patients’ data published between the beginning of January 2020 and the end of December 2021. Excluded from data analysis were reviews, abstracts, proceedings, and editorials. Cohort studies which did not provide sufficient individual data were also excluded.

## Results

Altogether 15 articles meeting the search criteria were included (Table 1).^4^^-^^17^ These 15 articles reported altogether 17 patients, 10 males and seven females. Age of these patients ranged from 20 to 75y (Table 1). Fourteen patients experienced pituitary compromise during a SARS-CoV-2 infection, one patient a pituitary complication after a SARS-CoV-2 vaccination, and two patients’ pituitary involvement in long-COVID syndrome (Table 1). Three patients experienced hypophysitis and 14 patients’ pituitary apoplexy. A pituitary adenoma was found in 13 patients (Table 1). The outcome was favourable in 15 patients but fatal in two.

**Table 1:** Patients with COVID-19 or side effects to SARS-CoV-2 vaccinations manifesting in the pituitary gland reported until December 2021.

Age	Sex	LOCN	Diagnosis	Treatment	Outcome	Reference
**SARS-CoV-2 infection**						
74	F	7d	Pituitary apoplexy^a^	Resection	Favourable	[4]
27	M	14d	Hypophysitis	Steroids	Favourable	[5]
55	M	6d	Pituitary apoplexy^a^	Resection	Fatal	[6]
54	F	0d	Pituitary apoplexy^a^	Resection	Favourable	[7]
56	M	NR	Pituitary apoplexy^a^	Resection	Favourable	[7]
53	M	NR	Pituitary apoplexy^a^	Resection	Favourable	[7]
65	F	NR	Pituitary apoplexy^a^	Decadron	Favourable	[8]
46	M	10d	Pituitary apoplexy^a^	Steroids	Favourable	[9]
35	M	4d	Pituitary apoplexy^a^	Antibiotics	Favourable	[10]
44	F	3d	Pituitary apoplexy^a^	Hydrocortisone	Favourable	[11]
28	F	NR	Pituitary apoplexy^b^	None	Favourable	[12]
27	M	NR	Pituitary apoplexy^a^	Ventilation	Fatal	[13]
20	F		Pituitary apoplexy^a^	Dexamethason, surgery	Favourable	[14]
47	M	NR	Pituitary apoplexy^a^	Resection	Favourable	[15]
**SARS-CoV-2 vaccination**						
51	M	2d^c^	Hypophysitis	Steroids, l-thyroxin	Favourable	[3]
**Long-COVID**						
75	M	42d	Pituitary apoplexy^a^	Hydrocortisone	Favourable	[16]
60	F	56d	Hypophysitis	Desmopressin	Favourable	[17]

LOCN, Latency between onset of COVID-19 and onset of neurological symptoms; NR, Not Reported.

a With pituitary adenoma.

b Adenoma disappeared presumably due to apoplexy during the SARS-CoV-2 infection.

c After second Moderna dose.

## Discussion

Affection of the pituitary gland due an infection with SARS-CoV-2 has been repeatedly reported (Table 1). The hypothalamus and pituitary glands are putative targets for SARS-CoV-2 due to the expression of Angiotensin-Converting Enzyme-2 (ACE-2) receptors on the surface of their cells.^18^ Several studies in humans and animals showed a significant ACE2 mRNA expression in hypothalamus and pituitary cells.^19^ Moreover, higher mortality and poorer outcomes have been described in COVID-19 patients with obesity, diabetes, and vertebral fractures, which are all highly prevalent in subjects with pituitary dysfunctions.^19^ This review provides evidence that apoplexy of pre-existing pituitary adenoma can be a complication of COVID-19.

Only a single patient with hypophysitis 2d after the second dose of the Moderna vaccine has been reported.^3^ The patient manifested with secondary adrenal insufficiency (hyponatriemia), central hypothyroidism, and central hypogonadism.^3^ The patient profited from steroids and substitution with l-thyroxin.^3^ Hypophysitis could be autoimmune as several other autoimmune disorders triggered by SARS-CoV-2 vaccination have been reported.

Long-COVID includes subacute COVID-19 (symptoms last 5‒11 weeks after the infection) and post-COVID syndrome (symptoms last >11 weeks). Frequent manifestations of long-COVID include tiredness, exhaustion, exercise intolerance, headache, dyspnoea, hyposmia, hypogeusia, muscle weakness, myalgia, impaired concentration, memory impairment, depression, anxiety disorder, insomnia, hair loss, angina chest pain, palpitations, ectopic beats, myocarditis, diabetes, and thromboembolism. Since at least some of these manifestations can be attributed to hypopituitarism, it is conceivable that at least some cases of post-COVID syndrome are in fact attributable to hypopituitarism. Long-COVID may also manifest as infundibulo-neuro-hypophysitis, as reported in a 60yo female 56d after COVID-19.^17^ This patient presented with central diabetes insipidus but without involvement of the anterior pituitary as demonstrated by normal hormone values.^17^ A second patient, a 75yo male developed pituitary apoplexy 42d after COVID-19. He recovered upon hydrocortisone and l-thyroxine.^20^ There was no need for immediate neurosurgical intervention.^20^ A further argument in favour of pituitary gland involvement in long-COVID is a study of 61 survivors of severe COVID-19 prospectively investigated for hormonal derangement three months after recovery. It was found that 24 patients had evidence of hypocorticism.^20^ Hypocorticism was transient and attributed to post-infectious hypophysitis.^20^

## Conclusions

This review shows that the pituitary gland can be involved in SARS-CoV-2 infections and can be a target of side effects to SARS-CoV-2 vaccinations and of long-COVID. Patients with a previous pituitary adenoma seem to be particularly at risk of suffering apoplexy of the pituitary gland from the SARS-CoV-2 infection. Hypopituitarism could play a role in the pathophysiology of long-COVID syndrome.

## Author's contribution

JF: Design, literature search, discussion, first draft, critical comments; FS: Literature search, discussion, critical comments, final approval.

## Ethics approval and consent to participate

Not applicable.

## Consent for publication

Not applicable.

## Availability of data and material

All data reported are available from the corresponding author.

## Funding

None received.

## Declaration of Competing Interest

The authors declare no conflicts of interest.
